# Editing the duplicated insulin-like growth factor binding protein-2b gene in rainbow trout (*Oncorhynchus mykiss*)

**DOI:** 10.1038/s41598-018-34326-6

**Published:** 2018-10-30

**Authors:** Beth M. Cleveland, Ginnosuke Yamaguchi, Lisa M. Radler, Munetaka Shimizu

**Affiliations:** 1 National Center for Cool and Cold Water Aquaculture, Agricultural Research Service, United States Department of Agriculture, Kearneysville, West Virginia United States of America; 2 Graduate School of Environmental Science, Hokkaido University, Sapporo Hokkaido, Japan; 3 Faculty of Fisheries Sciences, Hokkaido University, Hakodate Hokkaido, Japan

## Abstract

In salmonids, the majority of circulating insulin-like growth factor-I (IGF-I) is bound to IGF binding proteins (IGFBP), with IGFBP-2b being the most abundant in circulation. We used CRISPR/Cas9 methodology to disrupt expression of a functional IGFBP-2b protein by co-targeting for gene editing IGFBP-2b1 and IGFBP-2b2 subtypes, which represent salmonid-specific gene duplicates. Twenty-four rainbow trout were produced with mutations in the IGFBP-2b1 and IGFBP-2b2 genes. Mutant fish exhibited between 8–100% and 2–83% gene disruption for IGFBP-2b1 and IGFBP-2b2, respectively, with a positive correlation (*P* < 0.001) in gene mutation rate between individual fish. Analysis of IGFBP-2b protein indicated reductions in plasma IGFBP-2b abundance to between 0.04–0.96-fold of control levels. Plasma IGF-I, body weight, and fork length were reduced in mutants at 8 and 10 months post-hatch, which supports that IGFBP-2b is significant for carrying IGF-I. Despite reduced plasma IGF-I and IGFBP-2b in mutants, growth retardation in mutants was less severe between 10 and 12 months post-hatch (*P* < 0.05), suggesting a compensatory growth response occurred. These findings indicate that gene editing using CRISPR/Cas9 and ligand blotting is a feasible approach for characterizing protein-level functions of duplicated IGFBP genes in salmonids and is useful to unravel IGF-related endocrine mechanisms.

## Introduction

The growth hormone (GH) – insulin-like growth factor (IGF)-I axis is a positive regulator of growth in vertebrates. It is understood that GH stimulates hepatic production and release of IGF-I into systemic circulation in both mammals^1^ and fish, thereby classifying this system as a major endocrine mechanism, although local production of IGF-I and IGF-II is increasingly recognized for its significance^2–4^. Insulin-like growth factor-I is widely recognized for its ability to stimulate growth-promoting mechanisms in muscle^5–7^ and bone^8,9^. Central to the effects of IGF-I are IGF binding proteins (IGFBP) that are essential for prolonging the half-life of IGF-I in circulation and regulating the availability of IGFs to target specific tissues^10,11^. In humans, less than 1% of circulating IGF-I is free and unbound to IGFBPs^12^. Six types of IGFBPs have been identified in human circulation, with IGFBP-3 being the major carrier of circulating IGFs^11,13^. This GH-IGF-I-IGFBP system is fully operative in teleosts^2,14,15^. However, specific to this group are two paralogs for each member of six IGFBPs except IGFBP-4 due to the lineage-specific whole genome duplication in the common teleost ancestor^16^. In addition, salmonids have between 19–22 IGFBP genes due to an ancestral salmonid-specific whole genome duplication event, in addition to duplicates of IGF-I and IGF-II in some lineages^17–19^. However, only two IGFBP family members (IGFBP-1 and IGFBP-2) contribute significantly to total IGF binding in plasma^15^.

Specific to these two family members are three major IGFBP subtypes, IGFBP-1a, IGFBP-1b, and IGFBP-2b that collectively bind greater than 99% of IGF-I in salmonid plasma^20^, and each is encoded by two salmonid specific gene duplicates (IGFBP-1a1/IGFBP-1a2; IGFBP-1b1/IGFBP-1b2 and IGFBP-2b1/IGFBP-2b2). Identification of these proteins from ligand binding assays is based on their molecular masses of 28–32, 20–25, and 40–45 kDa, for IGFBP-1a, -1b, and -2b, respectively^21,22^. The binding protein of greatest abundance in plasma is IGFBP-2b which is functionally homologous to IGFBP-3 in mammals, both binding approximately 80% of total circulating IGF-I^20,23^. Protein abundance and/or expression of IGFBP-2b decreases during feed deprivation and increases upon refeeding, following the directional regulation of plasma IGF-I abundance^24–27^. These expression patterns support that IGF-I and IGFBP-2b are co-regulated, perhaps to achieve a specific free-to-bound IGF ratio that promotes an appropriate physiological response. In contrast, the IGFBP-1 subtypes appear to be growth-inhibitory, as is the case in mammals, since they exhibit disparate expression patterns compared to IGFBP-2b by increasing during feed deprivation^28–31^. Although other IGFBP family members (IGFBP-3–6) are not detected in fish plasma, they are being increasingly recognized for IGF-independent roles and their significance at the local level for sequestering hepatic and locally-derived IGFs to peripheral tissues^10,32–34^.

Understanding the functional roles of the IGFBP subtypes will be critical to establish their specific roles as modulators of IGF signaling and loss-of-function studies are critical to identify these protein-level functions. Advancements in gene editing technology, particularly using the Clustered Regularly Interspaced Short Palindromic Repeats (CRISPR)/Cas9 system, has expanded the capacity for targeted gene mutagenesis in many animals, including fish^35,36^. This technology has been successfully performed in several aquacultured species, including Atlantic salmon^37,38^, catfish^39,40^, tilapia^41,42^, and carp^43,44^ to induce a range of phenotypes related to fertility, muscle growth, and disease resistance. In Atlantic salmon the CRISPR/Cas9 system is efficient at inducing bi-allelic mutations in the F0 generation; although both homozygous and heterozygous mutants are produced that result in a proportion of individuals displaying a mosaic phenotype^37,38^. While production of an F1 population can resolve the issue of mosaics, this is a challenge for fish with long generation times or sterile phenotypes. Another challenge is that since salmonids have multiple subtypes of a single gene, double knockout may be critical to analyze loss-of-function.

Given the role of IGFBP-2b as the major carrier of IGF-I in salmonid plasma, our objective was to target the two IGFBP-2b subtypes, IGFBP-2b1 and IGFBP-2b2, for gene editing using the CRISPR/Cas9 system in rainbow trout. We describe production of rainbow trout with mutations in both IGFBP-2b subtypes and a subsequent reduction in plasma IGFBP-2b that is proportional to the extent of gene mutagenesis. These findings indicate that gene editing by CRISPR/Cas9 in rainbow trout is a feasible approach for disrupting expression of functional proteins of duplicated genes and is a valuable tool for disentangling the role of IGFBPs as regulators of IGF signaling.

## Results

**Figure 1:**
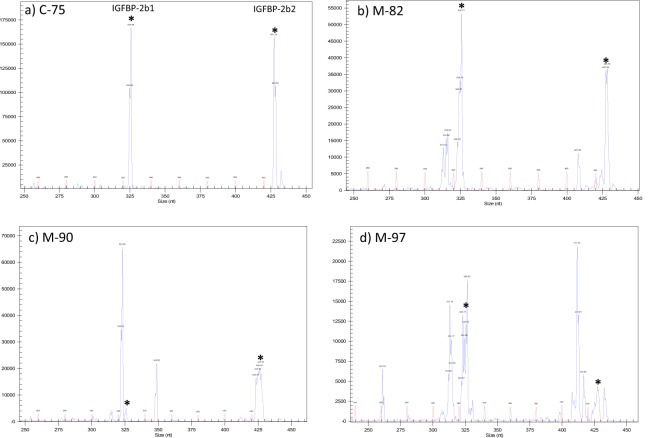
Representative chromatograms generated by PCR followed by amplicon separation by capillary gene electrophoresis. Each panel represents a different fish; sample numbers are shown that correspond to numbers in Table 1. Fish #C-75 (panel a) is identified as a control while fish in panels b–d are identified as mutants. Blue chromatograms represent amplicons while the red chromatogram is the base pair ladder. Peak height is directly proportional to amplicon abundance. The intact gene product for IGFBP-2b1 is 326 bp (left-most product, calculated size relative to bp ladder: ~325.8 bp) and 428 bp for IGFBP-2b2 (right-side product, calculated size: 427.3 bp). Asterisks are placed above the peaks corresponding to the intact gene products.

**Figure 2:**
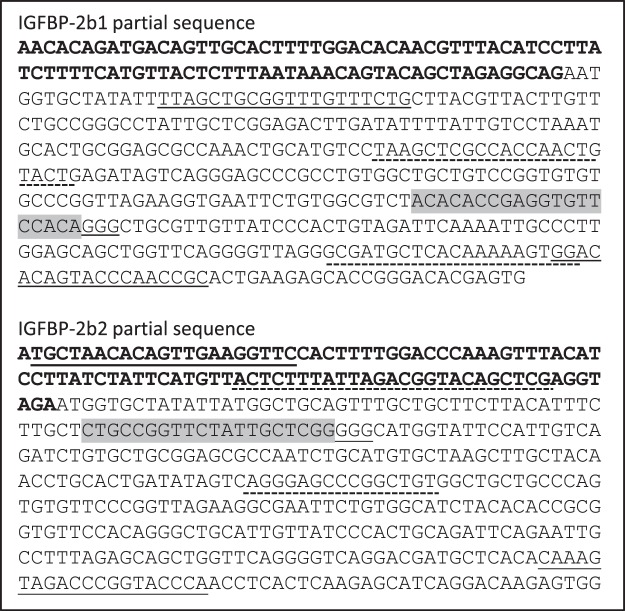
Genomic DNA sequence of the first exon and the upstream intron region within the IGFBP-2b1 and IGFBP-2b genes. The exon is in standard text while the intron is bold. The target crRNA annealing regions are highlighted in gray and the adjacent PAM site is underlined. Primer annealing sites for detection of indels by PCR are shown with the solid underline. Primer annealing sites for sequencing are shown with the dashed underline.

**Figure 3:**
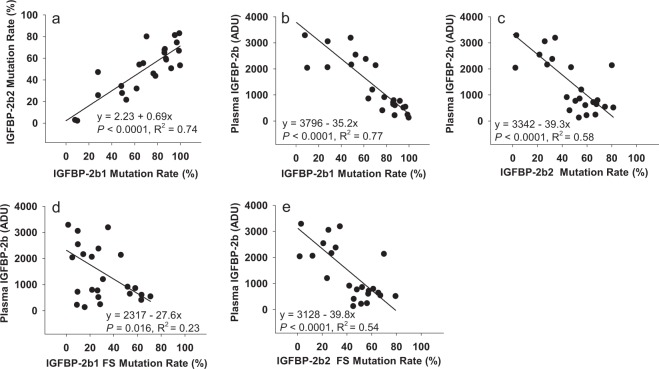
Scatterplots indicating the relationship between gene mutation rates and plasma IGFBP-2b abundance among the mutant fish. Results of the linear regression analyses are shown within each graph. ADU: arbitrary density units.

Fertilized rainbow trout eggs (embryos) were injected with ribonucleotide complexes (gRNA + Cas9, described in the Materials and Methods section) between 2–7 hours post-fertilization that target the IGFBP-2b1 and IGFBP-2b2 genes. Injected embryos and untreated embryos were hatched separately and maintained as separate lots through first-feeding, after which 35 fry from injected embryos were combined with untreated fish in a single tank to avoid tank effects on growth. At 7 months post-hatch fish were tagged with passive integrated transponders (PIT) and a fin clip was collected. Genomic DNA was isolated from fin clips and PCR followed by capillary gel electrophoresis was performed to distinguish between fish with intact genes (controls) and fish exhibiting gene mutagenesis (mutants). Twenty four candidate mutant fish were identified from this initial PCR screen. Representative chromatograms from a control (#C-75) and fish identified as mutants (M-82, M-90, M-97) are presented in Fig. 1. The expected IGFBP-2b1 and IGFBP-2b2 amplicons, without indels, are 326 and 428 base pairs, respectively (Figs 1 and 2). Indels are clearly discernable in Fig. 1 by multiple peaks near expected amplicons for Fish #M-82, M-90, and M-97, thereby classifying these fish as mutants. Fish #C-75 indicates single amplicons at the expected length and is therefore characterized as a control. Amplicon chromatograms for all fish subsequently identified as mutants and a subset of controls analyzed for plasma IGF-I and IGFBP-2b abundance are shown in Supplementary Fig. S1. The sequence and frequency of IGFBP-2b1 and IGFBP-2b2 gene variants were characterized for all mutant fish and a single control fish (#C-75). A summary of total reads, mutant reads, and mutant reads predicted to cause a frameshift mutation are shown in Table 1. For IGFBP-2b1, the percent of mutant reads for individual fish ranged between 7.88–99.92% (average: 69%) of total reads, with between 1.64–70.97% (average: 32%) of total reads causing frameshift mutations. For IGFBP-2b2, the percent of mutant reads ranged between 2.15–82.52% (average: 50%) of total reads, with between 2.26–68.97% (average: 43%) of total reads causing frameshift mutations. A strong correlation between mutation rates of each gene (Fig. 3a, R^2^ = 0.74, *P* < 0.0001) suggests that delivery of gRNA and Cas9 reagents into the embryo is rate-limiting for gene editing efficacy.

**Table 1:** Summary of total and mutant reads for IGFBP-2b1 and IGFBP-2b2 for a single control (#C-75) and each mutant fish.

Fish #	IGFBP-2b1					IGFBP-2b2					Average Mutant %	Average Frame-shift %	IGFBP-2b Abundance (ADU)
	Target Reads	Mutant Reads	Mutant %	Frameshift Mutant Reads	Frame-shift %	Target Reads	Mutant Reads	Mutant %	Frameshift Mutant Reads	Frame-shift %			
C-75	102205	439	0.43	208	0.20	73599	315	0.43	315	0.43	0.43	0.32	4770.6
M-38	130384	12820	9.83	6658	5.11	75747	1630	2.15	1292	1.71	5.99	3.41	2040.0
M-39	106473	93191	87.53	67537	63.43	93341	54711	58.61	53338	57.14	73.07	60.29	606.9
M-42	117540	82728	70.38	54061	45.99	99031	79321	80.10	69191	69.87	75.24	57.93	2138.1
M-45	100114	86525	86.43	21788	21.76	112178	76858	68.51	68638	61.19	77.47	41.48	796.9
M-46	110024	57975	52.69	10726	9.75	94621	20416	21.58	19719	20.84	37.14	15.30	2543.8
M-47	105896	105599	99.72	16436	15.52	107615	57443	53.38	48417	44.99	76.55	30.26	132.1
M-48	116452	71837	61.69	31584	27.12	114099	36394	31.90	35148	30.80	46.80	28.96	2379.6
M-49	98969	98887	99.92	70078	70.81	81599	67339	82.52	47132	57.76	91.22	64.29	NA
M-56	94822	81564	86.02	9052	9.55	92340	60122	65.11	53173	57.58	75.57	33.57	720.2
M-59	119493	57773	48.35	42019	35.16	94932	32508	34.24	32508	34.24	41.30	34.70	3192.6
M-62	106914	93317	87.28	9637	9.01	118782	70794	59.60	61009	51.36	73.44	30.19	220.7
M-63	106686	81489	76.38	67488	63.26	93837	43035	45.86	42660	45.46	61.12	54.36	407.5
M-64	89830	43978	48.96	12900	14.36	108598	30162	27.77	29792	27.43	38.37	20.90	2163.1
M-73	71027	5598	7.88	1166	1.64	94429	2812	2.98	2708	2.87	5.43	2.26	3289.9
M-82	88604	56730	64.03	51520	58.15	103346	55836	54.03	53925	52.18	59.03	55.17	860.9
M-89	95525	26763	28.02	9263	9.70	122216	31536	25.80	30544	24.99	26.91	17.35	3057.2
M-90	109703	104315	95.09	29700	27.07	109751	89246	81.32	86991	79.26	88.21	53.17	516.4
M-93	86706	58523	67.50	26839	30.95	94676	52436	55.38	22596	23.87	61.44	27.41	1206.1
M-95	100239	27951	27.88	21601	21.55	90964	42868	47.13	11029	12.12	37.51	16.84	2062.3
M-96	111179	95667	86.05	59567	53.58	101967	68983	67.65	66613	65.33	76.85	59.46	644.5
M-97	80600	78020	96.80	57203	70.97	73712	54927	74.52	49361	66.96	85.66	68.97	545.5
M-106	71828	70874	98.67	20493	28.53	107918	72139	66.85	60598	56.15	82.76	42.34	243.9
M-107	87747	68541	78.11	45343	51.67	85947	37473	43.60	35938	41.81	60.86	46.74	916.1
M-108	96546	88737	91.91	25460	26.37	112393	56845	50.58	53891	47.95	71.25	37.16	772.1

The number and percent reads that result in frameshift mutations are also indicated. Averaged across all samples, 99.7% of the filtered reads from the IGFBP-2b1 PCR mapped to the IGFBP-2b1 reference sequence (range: 96.5%–99.9%). Similarly, 99.86% of the filtered reads from the IGFBP-2b2 PCR mapped to the IGFBP-2b2 target reference (range: 98.5%–99.9%). ADU: arbitrary density units.

Fish #90 is shown as an example of the gene variants present at a frequency greater than 1% of total reads (Table 2). The most frequent gene variant for IGFBP-2b1 exhibited a three base pair deletion (60%), followed by a 26 base pair insertion (17%), the reference sequence (5%), an 11 base pair deletion (4%), a 12 base pair deletion (2%), a 31 base pair deletion (1.5%) and a six base pair deletion (1%). These gene variants correlate to the IGFBP-2b1 amplicon peaks for Fish #M-90 (Fig. 1c) identifying the presence and relative abundance of major indels. The identity and frequency of gene variants for IGFBP-2b2 in Fish #M-90 also reflected the population of indels in the amplicon chromatogram (Fig. 1c).

**Table 2:** Gene variants detected in Fish #M-90 at greater than 1% frequency.

Fish #M-90: Gene Variants	Type	Percent of Total Reads	Indel Length
IGFBP-2b1			
TCTGTGGCGTCTACACACCGAGGTGTT–CAGGGCTGC	Deletion	60.26	−3
TCTGTGGCGTCTACACACCGAGGTGTG**tagacgacagggctgcgttgtcacac**CGACAGGGCTGC	Insertion	16.57	26
TCTGTGGCGTCTACACACCGAGGTGTTCCACAGGGCTGC	Ref Seq	4.65	0
TCTGTGGCGTCTACACACCGAGGT-----------CTGC	Deletion	4.14	−11
TCTGTGGCGTCTACACAC------------CAGGGCTGC	Deletion	1.63	−12
TCTGTG-------------------------------GC	Deletion	1.44	−31
TCTGTGGCGTCTACACACCGAGGTGT------GGGCTGC	Deletion	1.18	−6
**IGFBP-2b2**			
ACATTTCTTGCTCTGCCGGTTCTATT----GGGGGCATGG	Deletion	29.05	−4
ACATTTCTTGCTCTGCCGGTTCTATTG–CGGGGGCATGG	Deletion	20.90	−2
ACATTTCTTGCTCTGCCGGTTCTATTGC-AGGGGGCATGG	Deletion	16.25	−1
ACATTTCTTGCTCTGCCGGTTCTATTGCTCGGGGGCATGG	Ref Seq	13.31	0
ACATTTCTTGCTCTGCCGGTTCTATT--------GCATGG	Deletion	7.89	−8
ACATTTCTTGCTCTGCCGGTTCTATTGC**AT**GG**T**GGCATGG	3 Base Changes	3.62	0
ACATTTCTTGCTCTGCCGGTTCTATTGCT–GGGCATGG	Deletion	1.94	−3
ACATTTCTTGCTCTGCCGGTTCTATTGC**AT**GGGGGCATGG	2 Base Changes	1.09	0

Bold indicate insertions and base pair substitutions compared to the reference sequence (Ref Seq). The crRNA target annealing site is underlined.

**Figure 4:**
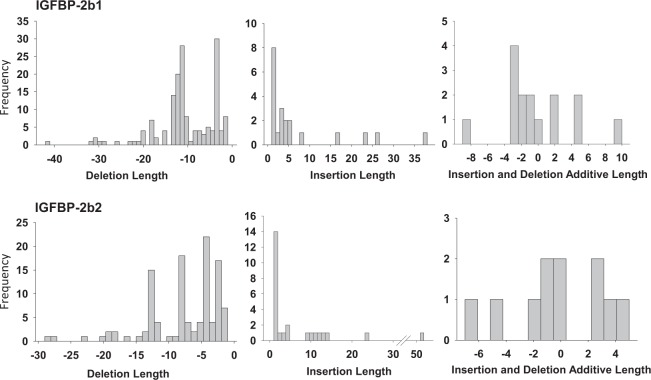
Histograms representing the frequency (count) of indel lengths. Only indels of greater than 1% abundance in any single fish were pooled for frequency data.

The frequencies of indel lengths for gene variants pooled from all mutants with greater than 1% frequency for any one fish are shown in Fig. 4. For IGFBP-2b1, deletions collectively ranged from −1 to −42 base pairs, with −3 and −11 deletions as the most frequent deletion length. Insertion length ranged from +1 to +38 base pairs, with +1 base pair as the most frequent. Gene variants were also characterized by combined insertions and deletions, with additive indel lengths that ranged from −9 to +9 base pairs, with −3 base pairs as the most abundant. Similar indel ranges were detected for IGFBP-2b2 variants (Fig. 4) although the most frequent deletion differed; the most abundant deletion and insertion were −4 and +1 base pairs, respectively. For both genes, deletions occurred with greater frequency than insertions.

**Figure 5:**
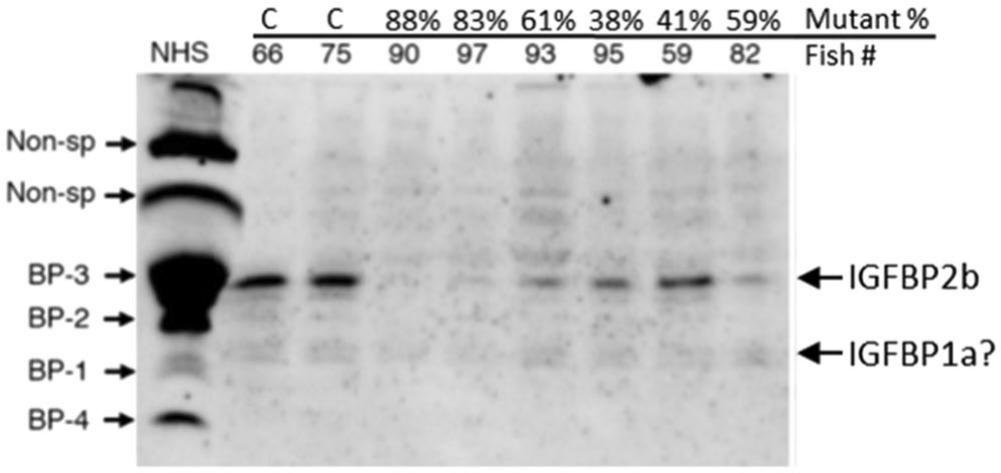
Representative ligand blot exhibiting the reduction in rainbow trout IGFBP-2b abundance with gene mutagenesis. Fish numbers are shown with the percent mutation rate averaged for IGFBP-2b1 and IGFBP-2b2. The IGFBP identities for normal human serum (NHS) are shown to the left. Non-sp: non-specific band, BP: IGF binding protein, C: control fish.

Ligand binding assays using labeled IGF-I were performed to determine whether circulating IGFBP-2b abundance was affected by gene mutagenesis. A representative blot is presented in Fig. 5 while IGFBP-2b abundance for all mutants is presented in Table 1. Additional ligand blots are shown in Supplementary Fig. S2. There was a negative correlation between the mutation rate of both IGFBP-2b1 and IGFBP-2b2 and IGFBP-2b protein abundance (Fig. 3b,c), indicating that as the genes became more disrupted there was less plasma IGFBP-2b protein. Similarly, there was a negative correlation between the frameshift mutation rate of each gene and IGFBP-2b abundance (Fig. 3d,e).

**Figure 6:**
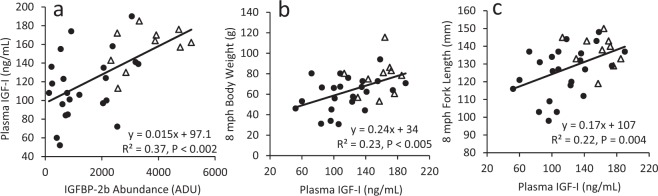
Linear regression analysis between plasma variables and indices of growth at 8 months post-hatch. Data for all mutant fish (n = 23) are presented as closed circles while control fish (n = 10) are presented as open triangles. The regression line is calculated from the collective data set (controls and mutants). ADU: arbitrary density units.

An analysis was performed to detect correlations between growth-related phenotypes and plasma IGF-I and IGFBP-2b abundance among all control and mutant fish (Fig. 6). A positive correlation between plasma IGF-I and IGFBP-2b abundance (Fig. 6a) suggests plasma IGF-I concentrations were reduced in mutants due to decreased IGFBP-2b abundance. Among all fish, there was a positive correlation between body weight, body length, and plasma IGF-I at 8 months post-hatch (Fig. 6b,c).

**Figure 7:**
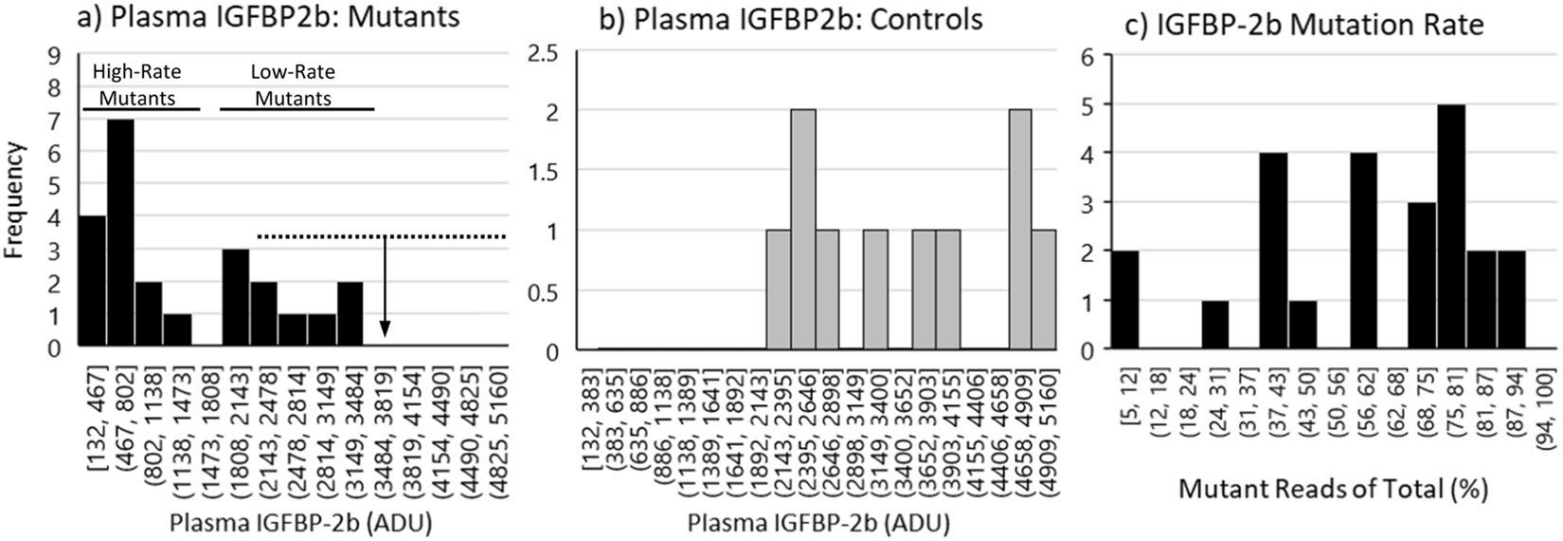
Histograms indicating the frequency distribution of the IGFBP-2b phenotype (panel a: mutants, panel b: controls) and mutant genotype (mutants: panel c). The solid horizontal lines represent the separation of the mutants into high-rate (<50% IGFBP-2b) and low-rate (>50% IGFBP-2b) mutant groups. The dashed horizontal line in panel a represents the range of IGFBP-2b values in control fish while the vertical arrow represents the control mean. The values on the x-axis represent the lower and upper boundary for each bin. ADU: arbitrary density units.

**Figure 8:**
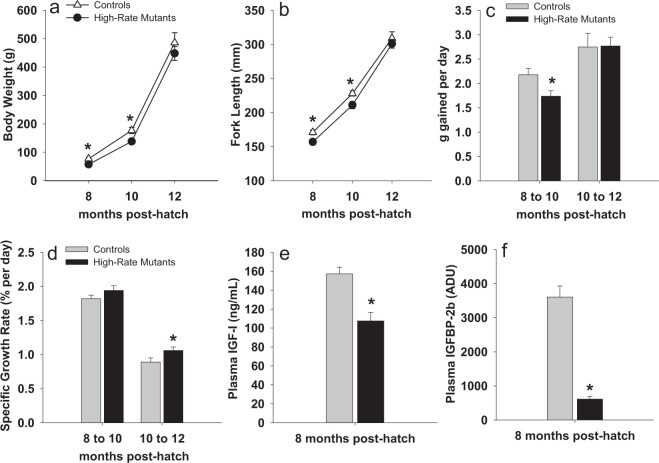
Growth-related performance and plasma IGF-I and IGFBP-2b abundance in controls (n = 10) and high-rate mutants (<50% IGFBP-2b, n = 14). In panels a and b, data for high-rate mutants are presented as closed circles while control fish are presented as open triangles. In panels (c–f), gray bars represent the control group while dark bars represent the high-rate mutant group. Bars indicate means ± SEM. Asterisks indicate a significant difference between groups, *P* < 0.05. ADU: arbitrary density units.

Plasma IGFBP2b abundance exhibited a bimodal distribution among mutants (Fig. 7a), with 14 of the 23 mutants exhibiting IGFBP-2b levels less than 50% of the control mean (Fig. 7a,b). The bimodal response is likely caused by the greater number of mutants with an average IGFBP-2b mutation rate of 50% or greater (Fig. 7c, 16 of 24 mutants). Although all mutants exhibited IGFBP-2b abundance values that were at least numerically lower than the control mean, the range of IGFBP-2b values in the control fish did overlap with the mutant treatment group (Fig. 7a,b). Due to the large variation in percent gene disruption that resulted in comparable variation in the IGFBP-2b abundance phenotype in the mutant treatment group, only the 14 mutant fish with plasma IGFBP-2b abundance lower than 50% of the control mean were used to detect effects of reduced IGFBP-2b on growth performance phenotypes. This sub-group, identified as high-rate mutants (<50% IGFBP-2b), exhibited a mean IGFBP-2b value of 613 ± 79 arbitrary density units (ADU) versus 3605 ± 328 ADU in the controls (Fig. 8f). The high-rate mutants also had reduced body weight and length at 8 and 10 months post-hatch, but not at 12 months post-hatch (Fig. 8a,b). Growth rates, calculated as g gained per day, were lower in high-rate mutants between 8 and 10 months but not between 10 and 12 months (Fig. 8c). However, specific growth rate (SGR) of high-rate mutants between 10 and 12 months post-hatch was greater than controls (Fig. 8d). Plasma IGF-I concentration was reduced in high-rate mutants compared to controls at both 8 months (Fig. 8e) and 10 months post-hatch (90.8 ng/mL versus 71.6 ng/mL, *P* < 0.05). At 8 months post-hatch the average IGF-I:IGFBP-2b ratio (ng/mL:ADU) was greater in high-rate mutants than in control fish (0.25 versus 0.05 ng/mL per ADU, *P* < 0.05).

## Discussion

Injecting gene-specific crRNA, tracrRNA, and Cas9 protein into fertilized rainbow trout embryos at the one-cell stage was successful at co-editing the IGFBP-2b1 and IGFBP-2b2 genes in rainbow trout, with a subsequent reduction in IGFBP-2b protein abundance in circulation to as low as 4% of control levels. Gene editing often results in a phenotypic gradient since F0 individuals are frequently heterozygous mutants^37,38^, which can provide limitations for interpreting autocrine and paracrine effects of gene mutagenesis. However, for endocrine-related proteins such as IGFBP-2b that are produced in liver and secreted into systemic circulation, having heterozygous mutants translated into a gradient of plasma IGFBP-2b abundance proportional to indel frequency. Achieving a range of protein knockdown provides an opportunity to assess the effect of reduced gene expression, mirroring the expression response to physiological perturbation in addition to determining effects of a near-absence of functional protein abundance.

Capacity for double knockout by gene editing is key to functional analyses of duplicated genes in salmonids. Since teleosts underwent a third round of whole genome duplication, they could have two subtypes of mammalian orthologs^45^. This is the case for the IGFBP family (e.g. IGFBP-2a and - 2b) except IGFBP-4^16^. The situation is more complex in salmonids where two copies of each IGFBP subtype gene (e.g. IGFBP-2b1 and -2b2) may be present due to an ancestral salmonid specific whole genome duplication event^17,19^. Thus, when IGFBP-2b1 is knocked out, IGFBP-2b2 may compensate its functions, making interpretation of knockout effects difficult. The result of the present study indicates the utility of the CRISPR/Cas9 system for co-editing duplicated genes in salmonids and warrants future functional analyses using this technique. Although it is also acknowledged that duplicated genes may have different functions, particularly those such as the IGFBP-2b paralogs with more divergent protein coding sequences. In these cases a single knockout would be valuable to isolate the function of each subtype. Due to limited egg availability, we were not able to independently target the IGFBP-2b subtypes to determine the relative contribution of each locus to total protein abundance. In addition, the close correlation between mutation rates of the two genes prevents the use of these data to make inferences regarding the relative contribution of each gene locus to total plasma IGFBP-2b abundance. Future studies that independently target IGFBP-2b1 and IGFBP-2b2 for gene editing will be valuable for clarifying this relationship.

A close correlation between gene mutation rates is consistent with previous reports that delivery of reagents into embryos is a rate-limiting step for gene editing efficiency; for this reason, salmonid researchers have co-targeted a phenotypic tracer gene as a screening tool for identifying mutants^37^. However, the correlation could also result if crRNAs are not able to distinguish between the two IGFBP-2b subtypes. The IGFBP-2b2 crRNA is not expected to mutate the IGFBP-2b1 gene since, in addition to having a two-base mismatch, the latter lacks the essential protospacer adjacent motif (PAM) site immediately upstream of the target region. However, the crRNA designed against IGFBP-2b1 may also edit IGFBP-2b2 since this gene only has a single mismatch at position 12 of the target sequence. This mismatch configuration did not significantly reduce gene editing activity in mammalian cell culture systems^46^. While single-base mismatches in the distal region (position #11–20) are better tolerated than the region adjacent to the PAM site (position #1–10), two or more mismatches generally disrupt cleavage activity^46,47^. Although this suggests crRNAs can anneal to off-target genes, cleavage requires a PAM site therefore BLAST analysis of candidate crRNAs will minimize the chances of off-target indels. Aligning the two crRNAs from the current study against the rainbow trout genome did not identify any genes, including other IGFBPs, with high likelihood of being unintended cleavage targets. We did not complete any genomic sequencing to identify possible off-target effects, although we acknowledge the possibility that undetected off-target mutations may have occurred.

A combination of the CRISPR/Cas9 system and ligand blotting using labeled IGF-I is a powerful tool to analyze how different degrees of IGFBP-2b mutation are reflected in actual protein abundance and how deletion of one type of IGFBP is compensated by other types. Ligand blotting using labeled IGF-I detects proteins with IGF-binding ability. It is not specific to a particular IGFBP but it is useful to detect different types of IGFBPs in plasma. The results of the present study showed that there was a good link between gene mutation rate and reduced protein abundance of IGFBP-2b, which suggests that the number of non-mutated cells producing IGFBP-2b was the primary determinant of its circulating level. In addition, ligand blotting revealed that there were no obvious or consistent compensatory responses by other IGFBPs in plasma to gene editing for IGFBP-2b, which is important information to interpret how IGF-I is maintained in the circulation without IGFBP-2b as discussed below.

The weaker correlation between IGFBP-2b1 frameshift mutation rate and protein abundance is initially suggestive of a reduced contribution compared to IGFBP-2b2 but arguments can be made against this concept. Transcriptomics data from Atlantic salmon support that hepatic *IGFBP-2b1* expression is approximately seven times greater than *IGFBP-2b2* expression^17^, suggesting that IGFBP-2b1 has a more dominant contribution to circulating protein. Additionally, approximately half of the base pair deletions in the IGFBP-2b1 gene did not result in frameshift mutations but it should not be assumed that this renders them ineffective at disrupting the functional protein. Loss of three and 12 base pairs will delete one and four amino acids, respectively, at the N-terminal region of IGFBP-2b within exon 1 that contains high-affinity IGF binding sites^16,48^ therefore the absence of key residues within this region could inhibit protein function or promote proteasome-mediated degradation due to misfolding.

The positive correlation between plasma IGF-I and IGFBP-2b abundance, in addition to reduced plasma IGF-I in high-rate mutants, supports that plasma IGF-I concentrations were regulated at least in part by reductions in IGFBP-2b. Increased body weight in control fish, that also had higher plasma IGF-I, during the early rearing phases is consistent with positive correlations between plasma IGF-I and body size in several fish species^49^, including rainbow trout^50^. Several studies in salmonids indicate co-directional regulation of IGF-I and plasma IGFBP-2b^25,51,52^, suggesting that maintaining an appropriate equilibrium between these proteins is significant for regulating a physiological response, particularly since the molar ratio of total IGF-I:IGFBP-2b in salmonid plasma is reported to be 1:1.5~2.8^51^. Therefore, the 80%+ reduction in plasma IGFBP-2b in the high-rate mutants, accompanied by only a 30% reduction in plasma IGF-I, suggests there may be greater concentrations of free IGF-I in mutant fish plasma. However, the absence of a consistent growth advantage in high-rate mutants is counter-intuitive to what would be expected from an increase in free IGF-I and/or the molar ratio of IGF-I:IGFBP-2b in plasma. It is possible that in these mutants, in addition to reduced plasma IGF-I, other IGF-related mechanisms in liver or peripheral tissues are further compensating for reductions in circulating IGFBP-2b. In salmonids, IGFBP-2b, IGFBP-1a, and IGFBP-1b are significant for transporting IGF-I in plasma^21^ but the ligand binding assay does not support compensatory regulation of the IGFBP-1 family in IGFBP-2b mutants. However, a compensatory response was reported in IGFBP-2 knockout mice, in which ligand binding revealed elevated serum IGFBP-1, IGFBP-3 and IGFBP-4^53^, indicating a physiological response to maintain IGF-I binding capacity. In addition to the major circulatory IGFBPs, the ability of IGF-I to induce signaling in peripheral tissues depends on its binding to IGF receptors at the cell surface, which is dependent on the abundance of surface receptors and local expression of IGFBPs that can both promote and inhibit ligand-receptor interactions^32,54^. Whether these mechanisms are regulated in mutants as a compensatory response to reduced IGFBP-2b protein remains to be determined but will be important for further characterizing the endocrine effects IGFBP-2b knockdown.

Data from the current study support that reduced IGFBP-2b abundance had a negative effect on body weight and length, at least during the early rearing periods (up to 10 months). These data are in agreement with a previous report of positive correlations between IGFBP-2b and individual growth rate in salmon^49^. Expression and abundance of IGFBP-2b in salmonids is under nutritional regulation, generally showing up-regulation with feeding, particularly following a fasting period^24,27,51,55^. Expression of *IGFBP-2b* is also under endocrine control; growth hormone, typically recognized for its anabolic effects, up-regulates IGFBP-2b^52,56^ which provides additional support for its positive effects on growth. However, in zebrafish over-expression of IGFBP-2 reduced embryonic growth^57^ and expression increased during fasting and decreased with GH treatment^58^, suggesting that functional roles of IGFBP-2 family proteins differ between species.

Despite initial reductions in body size and rate of gain in mutants, at 12 months post-hatch body weight, fork length, and weight gain did not differ between high-rate mutants and controls. Although these mutants exhibited faster SGR between 10 and 12 months of age, this response appears to primarily be a function of lower 10 month body weight in mutants rather than greater weight gain. This potential growth improvement suggests that effects of IGFBP-2b knock-down on body weight could depend on the period of development. Lending support to this concept are changes in IGFBP-2b abundance during smoltification in salmon, indicating developmental stage-specific expression patterns are important for physiological transitions^28,52^. While smoltification is not a relevant mechanism for fish in this study, it is known that muscle growth is driven by different mechanisms in early versus late growth stages. Juvenile muscle growth in salmonids is driven primarily by hyperplasia while hypertrophy is the dominant mechanism driving the increase in muscle mass in larger fish^59,60^. Subsequent analysis of growth-related phenotypes in mutants, including myogenic mechanisms and muscle fiber density, will be valuable for further characterizing the significance of this response.

In conclusion, IGFBP-2b1 and IGFBP-2b2 mutagenesis induced by gene editing reduced abundance of the IGFBP-2b protein in plasma in some individuals to levels less than 10% of control fish, indicating the value of this approach to unraveling IGF-related mechanisms in fish that have multiple subtypes of each component. These findings warrant additional studies to further characterize phenotypes in mutant fish, particularly regarding changes in the amount of free IGF-I and understanding how related mechanisms are affected in peripheral tissues during both basal growth and physiological perturbation. These studies are essential to understanding the GH-IGF axis that is central to regulation of numerous physiological processes in fish, including growth, nutrient metabolism, and osmoregulation. Our approach to combine the CRISPR/Cas9 system and ligand blotting was useful to link reduced expression to protein abundance of target IGFBP subtypes and capture possible compensatory responses of other IGFBPs.

## Materials and Methods

### Genetic modification procedures

Reagents for gene mutagenesis were purchased from Integrated DNA Technologies (IDT) from the Alt-R CRISPR-Cas9 System product line. crRNA sequences were designed using online programs (crisprscan.org, crispr.wustl.edu/crispr/index.html, crispr.med.harvard.edu/sgRNAScorer/) to target IGFBP-2b1 (NM_001124557.1) and IGFBP-2b2 (JX674936.1), and avoided sequence similarity with other genes, including other IGFBPs. To promote disruption of a functional protein, only the first exons were used to identify potential crRNA sequences. Intron/exon boundaries were determined by aligning the cDNA sequence against the gene sequence identified from the rainbow trout genome assembly^61^ (GenBank: GCA_002163495.1). The location of the crRNA target site within the first exon is shown in Fig. 2. Each crRNA and tracrRNA were diluted to 100 μM in the supplied IDTE buffer. The gRNA solution was produced by combining 1.5 μL of each crRNA, 3.0 μL tracrRNA, and 19.0 μL Duplex buffer. The solution was heated at 95 °C for 5 min. Cas9 protein was diluted to 0.5 μg/ μL in Cas9 working buffer (20 mM HEPES, 150 mM KCl, pH 7.5) and combined with an equal volume of gRNA solution. Ribonucleoprotein (RNP) complexes were assembled by incubating the gRNA + Cas9 mixture at 37 °C for 10 min. Phenol red was used to visualize RNP delivery.

All procedures involving animal use were approved by the National Center for Cool and Cold Water (NCCCWA) Institutional Animal Care and Use Committee (IACUC) (Protocols #098 and #115) and were carried out in accordance with Institutional animal care guidelines. Eggs and milt were collected from two-year old rainbow trout housed at the NCCCWA. Eggs were rinsed with milt activation solution (102.8 mM NaCl, 10 mM Tris, 20 mM glycine, pH 9.0, 1 mM reduced glutathione) prior to fertilization. The microinjection procedure was modified from procedures previously published for rainbow trout and Atlantic salmon^38,62^. Embryos (fertilized eggs) were held in milt activation solution at 10 °C and injected 2–7 hr post-fertilization. Immediately prior to injection, embryos were stabilized on a tray fixed within a Petri dish and submerged in 10 °C saline solution (0.9% NaCl). A handheld positive displacement microinjector was used to deliver between 100–200 nL of the RNP complex directly into the blastodisc. Injection was visualized using a stereomicroscope (Nikon SMZ1000). Once injected, embryos were transferred to spring water (10–12 °C) for hatching. Injected embryos and untreated embryos were hatched separately and maintained as separate lots through first-feeding, after which 35 fry from injected embryos were combined with untreated fish in a single tank to avoid tank effects on growth. The pooled fish were reared according to standard operating protocols until approximately 20 g (7 months post-hatch) when they were anesthetized with tricaine methanesulfonate (MS222, 100 mg/L), tagged with passive integrated transponders (PIT), and the adipose fin was removed and stored in ethanol for DNA analysis to differentiate between fish with disrupted (mutants) and intact (controls) IGFBP-2b genes (PCR described below). At 8 and 10 months post-hatch fish were again anesthetized and a 300 μL blood sample was removed from gill vasculature into a heparinized syringe to determine IGFBP-2b and IGF-I abundance. At 8, 10, and 12 months post-hatch, body weight and fork length were recorded.

### Mutant identification and characterization

Genomic DNA (gDNA) was isolated from individual fin clips using a standard gDNA isolation protocol with an overnight proteinase K digestion and isopropanol precipitation. The gDNA was used in a multiplex PCR that amplified the two regions containing the targeted crRNA annealing sites within the IGFBP-2b1 and IGFBP-2b2 genes (Fig. 2, Table 3). The PCR contained 10 ng gDNA, 375 nM each forward and reverse primer, 1.0 U Taq polymerase, and 1X reaction buffer with 250 µM dNTPs. The PCR reaction was as follows: 95 °C for 5 min and 30 cycles of 95 °C for 15 sec, 60 °C for 30 sec, and 72 °C for 30 sec. The reaction products (0.5 μL) were diluted with 38 μL running buffer and 0.25 μL size standard 600 (ABSciex) and separated using capillary gel electrophoresis with the BeckmanCoulter Genetic Analysis System. A single peak at 326 bp and 428 bp was assumed as an intact gene while the presence of multiple peaks indicates indels caused by gene mutagenesis. Representative images are provided in Fig. 1. Fish that exhibited indels were identified as mutants. A second PCR was completed for mutants and one control fish using sequencing primers (Fig. 2, Table 3); PCR amplicons were analyzed for gene variants through the Amplion-EZ service provided by GENEWIZ (South Plainfield, NJ). Briefly, the DNA amplicon was indexed and enriched by limited cycle PCR. The DNA library was validated using TapeStation (Agilent Technologies, Palo Alto, CA, USA), and was quantified using Qubit 2.0 Fluorometer and real time PCR (Applied Biosystems, Carlsbad, CA, USA). The DNA library was loaded on an Illumina instrument according to manufacturer’s instructions (Illumina, San Diego, CA, USA). Sequencing was performed using a 2 × 250 paired-end (PE) configuration; image analysis and base calling was conducted by the Control Software on the instrument. Before analysis, sequence reads were trimmed to remove possible adapters and nucleotides with poor quality at 3′ end. Then trimmed reads were aligned to the reference sequence and variant detection was performed using the GENEWIZ proprietary Amplicon-EZ program. Frameshift mutations were defined as the subset of the mutant reads with an additive indel length that was not a multiple of three.

**Table 3:** Primer sequences used for polymerase chain reactions.

Oligo	Sequence (5′-3′)	Product Size (bp)
**PCR Primers**		
IGFBP-2b1	For: TTAGCTGCGGTTTGTTTCTG	326
	Rev: GCGGTTGGGTACTGTGTCC	
IGFBP-2b2	For: TGCTAACACAGTTGAAGGTTC	428
	Rev: TGGGTACCGGGTCTACTTTG	
**Sequencing primers**		
IGFBP-2b1	For: TAAGCTCGCCACCAACTGTA	201
	Rev: TCCACTTTTTGTGAGCATCGC	
IGFBP-2b2	For: ACTCTTTATTAGACGGTACAGCTCG	200
	Rev: ACAGCCGGGCTCCCT	

### Phenotype analysis

Blood was collected using heparizined syringes and held on ice prior to processing (less than 2 hr). Plasma was separated by centrifugation (2000 cpm, 10 min, 4 °C) and stored at −80 °C for analysis of plasma IGF-I and IGFBP-2b abundance. Plasma IGF-I was quantified according to a previously published time-resolved fluoroimmunoassay^63^, with minor modifications, using recombinant trout IGF-I and anti-barramundi IGF-I antiserum (GroPep, South Adelaide, Australia). Western ligand blotting with digoxigenin-labeled human IGF-I (DIG-hIGF-I) was carried out as previously described^64^. After electroblotting, the nitrocellulose membrane was incubated with 10–50 ng/ml DIG-hIGF-I for 2 h at room temperature and then incubated with an antibody against DIG, conjugated with horseradish peroxidase (Roche, Indianapolis, IN) at a dilution of 1:1,500–2,500 for 1 h at room temperature. IGFBP was visualized by use of enhanced Chemiluminescence (ECL) Prime Western blotting reagents (Amersham Life Science, Arlington Heights, IL) and a luminescent image analyzer (LAS-1000 mini; Fuji Film, Tokyo Japan). Plasma IGFBP levels were semi-quantified by using Image-J 1.440^65^, normalized to the human IGFBP-4 band intensity and expressed as arbitrary density units (ADU).

### Statistics

Analyses were performed using PC-SAS (v 9.2) and SigmaPlot (v 11). Linear correlations between variables were determined by linear regression analysis. The Student’s t-test was used to detect differences between control and mutant group means. Data were tested for normality (Shapiro-Wilk) and correlations were determined as significant when *P* < 0.05. The datasets generated during and/or analyzed during the current study are available from the corresponding author on request.

## Electronic supplementary material

Supplementary Files
