# Supplementary material for: Editing the duplicated insulin-like growth factor binding protein-2b gene in rainbow trout (Oncorhynchus mykiss)
Source: Sci Rep. 2018 Oct 30;8:16054. doi: 10.1038/s41598-018-34326-6 (PMC6207780; doi:10.1038/s41598-018-34326-6)

Supplementary Figure S1

Manuscript Title: Editing the duplicated insulin-like growth factor binding protein-2b gene in rainbow trout (*Oncorhynchus mykiss*)

Authors: Beth M. Cleveland, Ginnosuke Yamaguchi, Lisa M. Radler, and Munetaka Shimizu

Supplementary Figure S1 Legend: Chromatograms generated by PCR followed by amplicon separation by capillary gene electrophoresis. Each panel represents a different fish; sample identifiers are at the top left of each chart. Blue chromatograms represent amplicons while the red chromatogram is the base pair ladder. Peak height is directly proportional to amplicon abundance. The intact gene product for IGFBP-2b1 is 326 bp (left-most product, calculated size relative to bp ladder: ~325.8 bp) and 428 bp for IGFBP-2b2 (right-side product, calculated size: 427.3 bp). Asterisks are placed above the peak corresponding to the intact gene product. Control (C), Mutant (M). Numbers indicate fish-specific identifier.

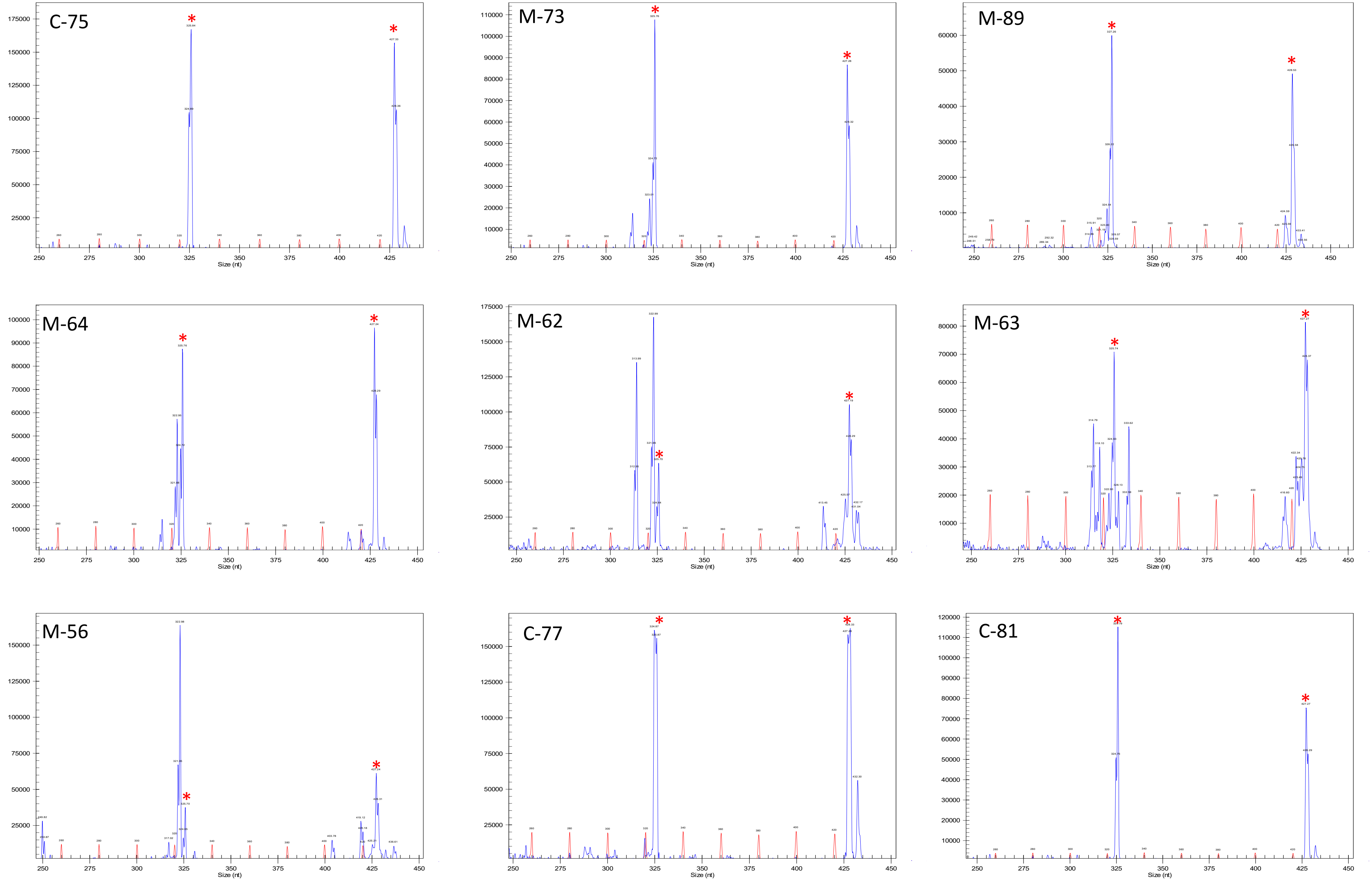

Supplementary Figure S1 (Continued)

Manuscript Title: Editing the duplicated insulin-like growth factor binding protein-2b gene in rainbow trout (*Oncorhynchus mykiss*)

Authors: Beth M. Cleveland, Ginnosuke Yamaguchi, Lisa M. Radler, and Munetaka Shimizu

Supplementary Figure S1 Legend: Chromatograms generated by PCR followed by amplicon separation by capillary gene electrophoresis. Each panel represents a different fish; sample identifiers are at the top left of each chart. Blue chromatograms represent amplicons while the red chromatogram is the base pair ladder. Peak height is directly proportional to amplicon abundance. The intact gene product for IGFBP-2b1 is 326 bp (left-most product, calculated size relative to bp ladder: ~325.8 bp) and 428 bp for IGFBP-2b2 (right-side product, calculated size: 427.3 bp). Asterisks are placed above the peak corresponding to the intact gene product. Control (C), Mutant (M). Numbers indicate fish-specific identifier.

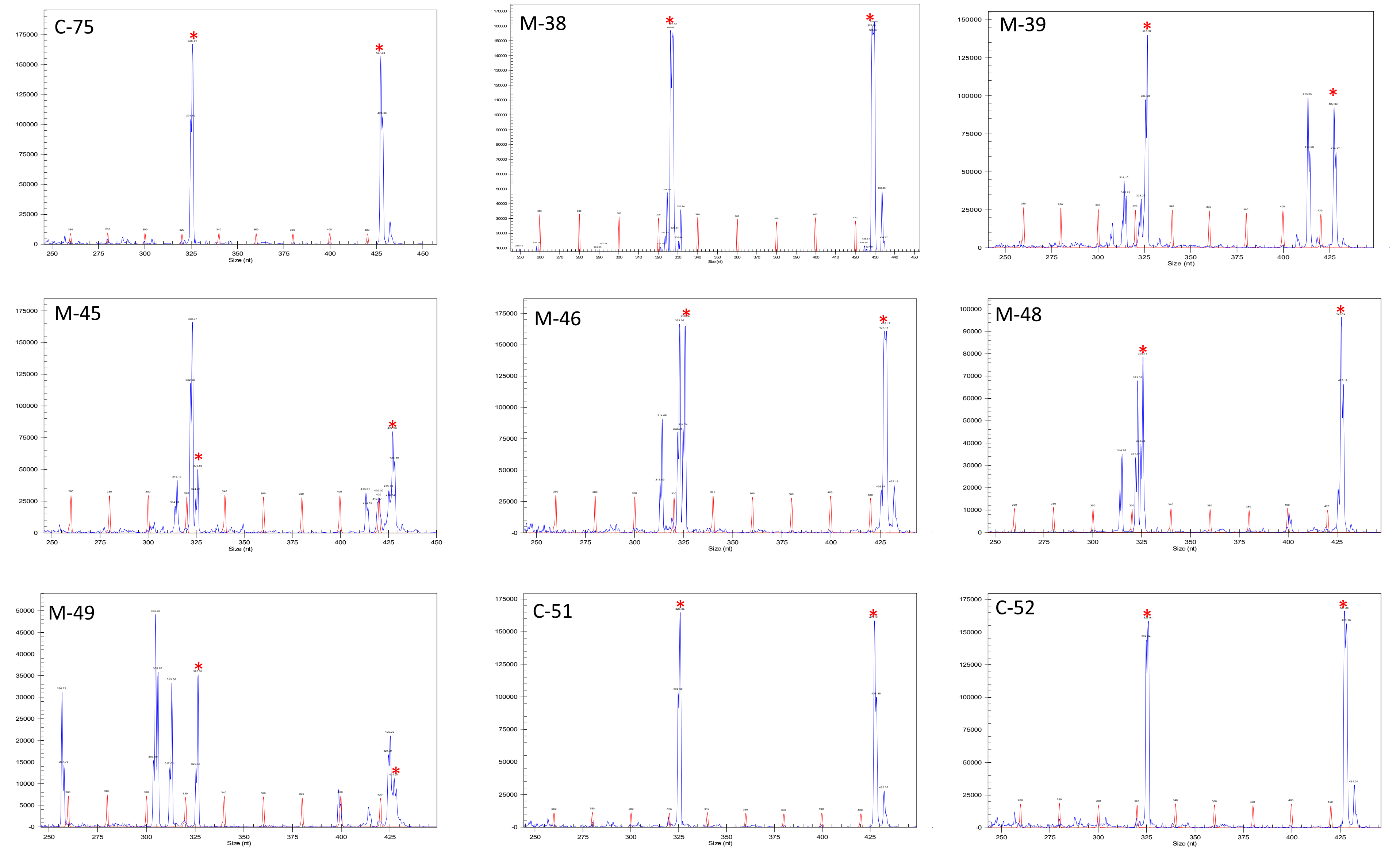

Supplementary Figure S1 (Continued)

Manuscript Title: Editing the duplicated insulin-like growth factor binding protein-2b gene in rainbow trout (*Oncorhynchus mykiss*)

Authors: Beth M. Cleveland, Ginnosuke Yamaguchi, Lisa M. Radler, and Munetaka Shimizu

Supplementary Figure S1 Legend: Chromatograms generated by PCR followed by amplicon separation by capillary gene electrophoresis. Each panel represents a different fish; sample identifiers are at the top left of each chart. Blue chromatograms represent amplicons while the red chromatogram is the base pair ladder. Peak height is directly proportional to amplicon abundance. The intact gene product for IGFBP-2b1 is 326 bp (left-most product, calculated size relative to bp ladder: ~325.8 bp) and 428 bp for IGFBP-2b2 (right-side product, calculated size: 427.3 bp). Red asterisks are placed above the peak corresponding to the intact gene product. Control (C), Mutant (M). Numbers indicate fish-specific identifier.

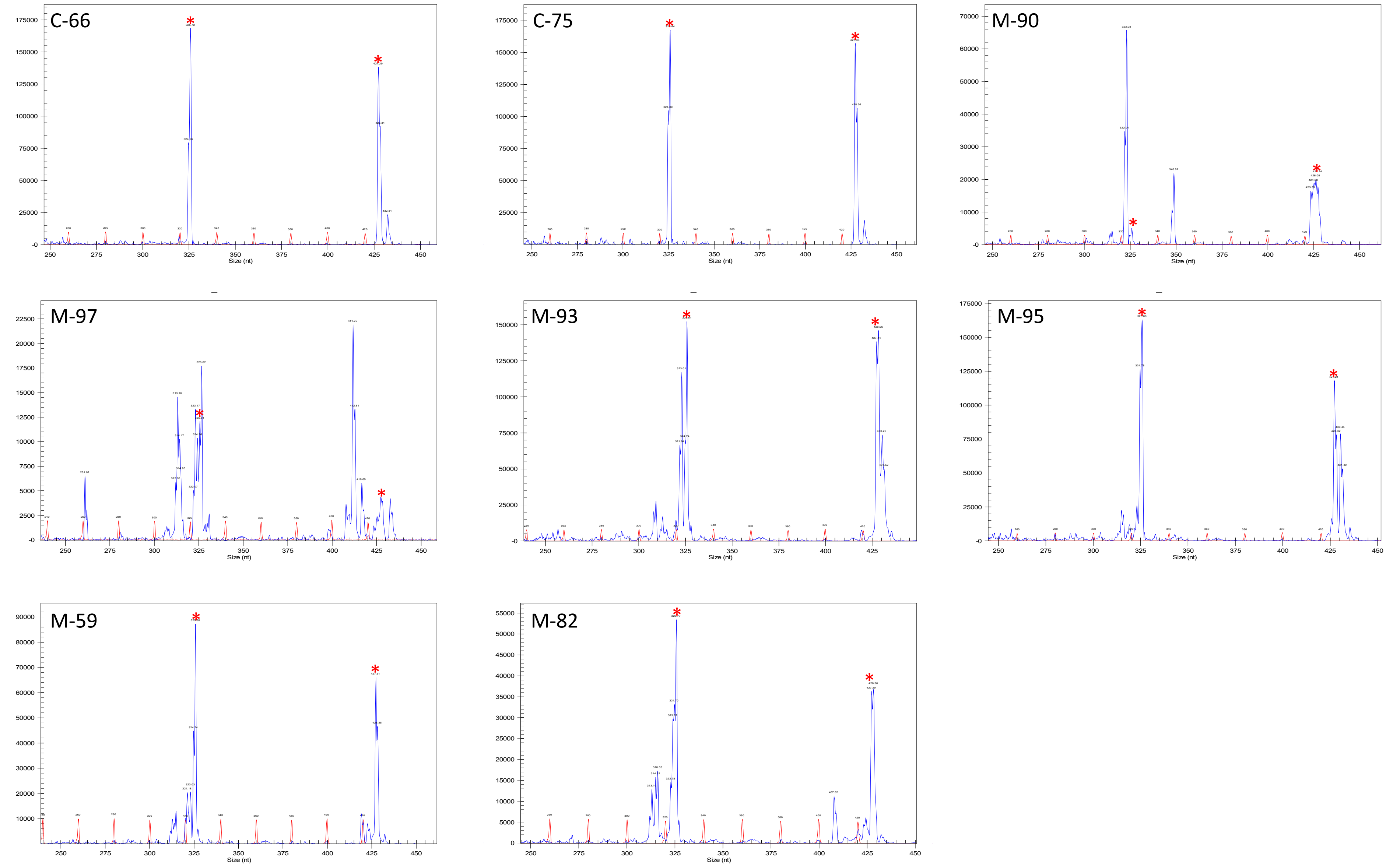

Supplementary Figure S1 (Continued)
Manuscript Title: Editing the duplicated insulin-like growth factor binding protein-2b gene in rainbow trout (*Oncorhynchus mykiss*)
Authors: Beth M. Cleveland, Ginnosuke Yamaguchi, Lisa M. Radler, and Munetaka Shimizu
Supplementary Figure S1 Legend: Chromatograms generated by PCR followed by amplicon separation by capillary gene electrophoresis. Each panel represents a different fish; sample identifiers are at the top left of each chart. Blue chromatograms represent amplicons while the red chromatogram is the base pair ladder. Peak height is directly proportional to amplicon abundance. The intact gene product for IGFBP-2b1 is 326 bp (left-most product, calculated size relative to bp ladder: ~325.8 bp) and 428 bp for IGFBP-2b2 (right-side product, calculated size: 427.3 bp). Red asterisks are placed above the peak corresponding to the intact gene product. The green asterisk above peaks at ~412 bp in samples C-103, 105, and 109 represent a gene variant with a 15 base pair deletion in the 5'UTR prior to exon 1. A PCR of these samples with internal sequencing primers yields a single intact product (shown in inset). Control (C), Mutant (M). Numbers indicate fish-specific identifier.

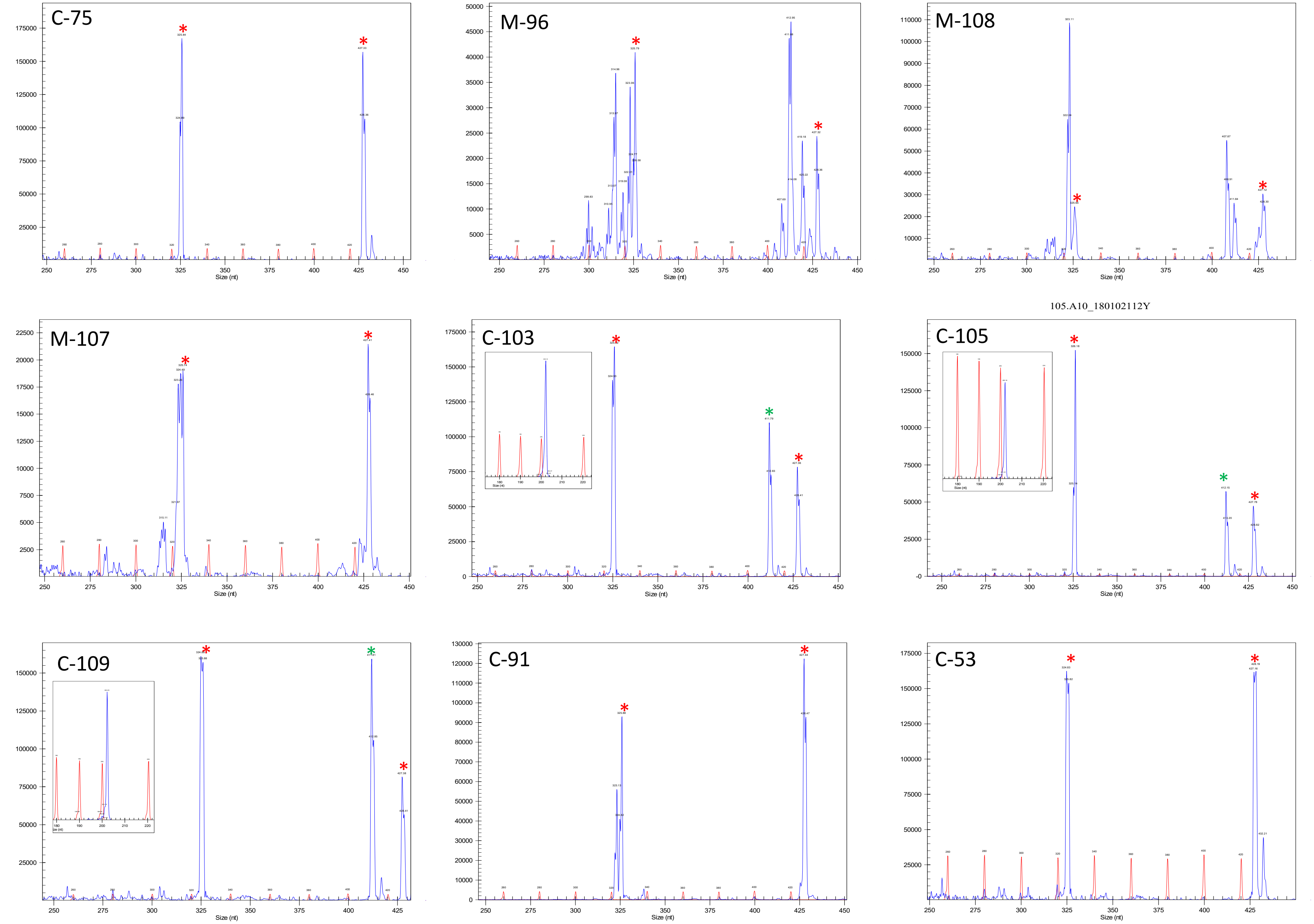

Supplementary Figure S1 (Continued)

Manuscript Title: Editing the duplicated insulin-like growth factor binding protein-2b gene in rainbow trout (*Oncorhynchus mykiss*)

Authors: Beth M. Cleveland, Ginnosuke Yamaguchi, Lisa M. Radler, and Munetaka Shimizu

Supplementary Figure S1 Legend: Chromatograms generated by PCR followed by amplicon separation by capillary gene electrophoresis. Each panel represents a different fish; sample identifiers are at the top left of each chart. Blue chromatograms represent amplicons while the red chromatogram is the base pair ladder. Peak height is directly proportional to amplicon abundance. The intact gene product for IGFBP-2b1 is 326 bp (left-most product, calculated size relative to bp ladder: ~325.8 bp) and 428 bp for IGFBP-2b2 (right-side product, calculated size: 427.3 bp). Asterisks are placed above the peak corresponding to the intact gene product. Control (C), Mutant (M). Numbers indicate fish-specific identifier.

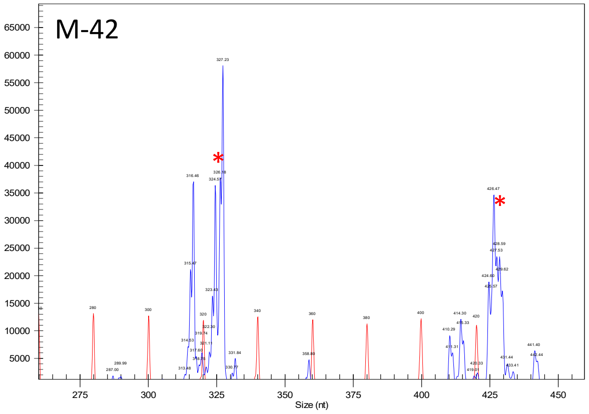

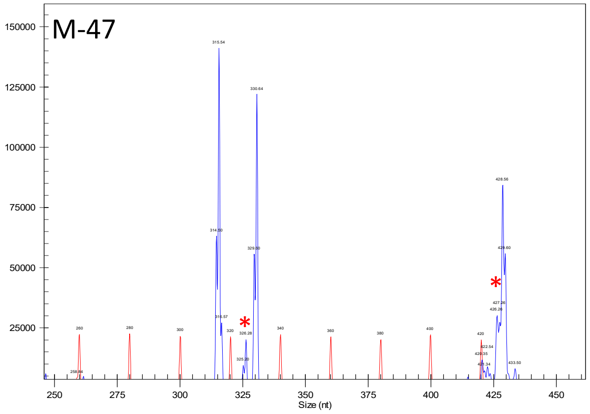

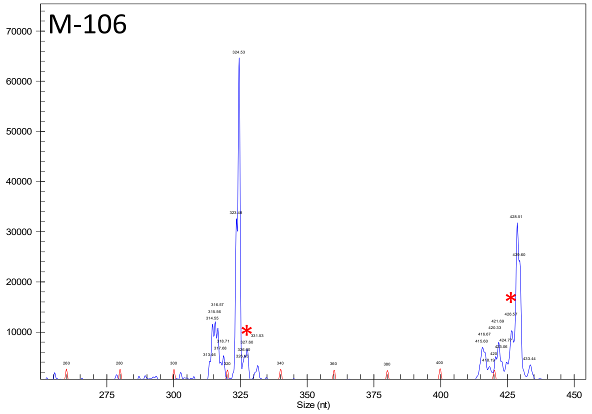

Supplementary Figure S2

Manuscript Title: Editing the duplicated insulin-like growth factor binding protein-2b gene in rainbow trout (*Oncorhynchus mykiss*)

Authors: Beth M. Cleveland, Ginnosuke Yamaguchi, Lisa M. Radler, and Munetaka Shimizu

Supplementary Figure S2 Legend: Ligand blots exhibiting reduction in rainbow trout IGFBP-2b abundance with increased gene mutagenesis. Fish numbers are shown with the percent mutation rate averaged for IGFBP-2b1 and IGFBP-2b2. The IGFBP identities for normal human serum (NHS) are shown to the left. Non-sp: non-specific band, BP: IGF binding protein, C: control

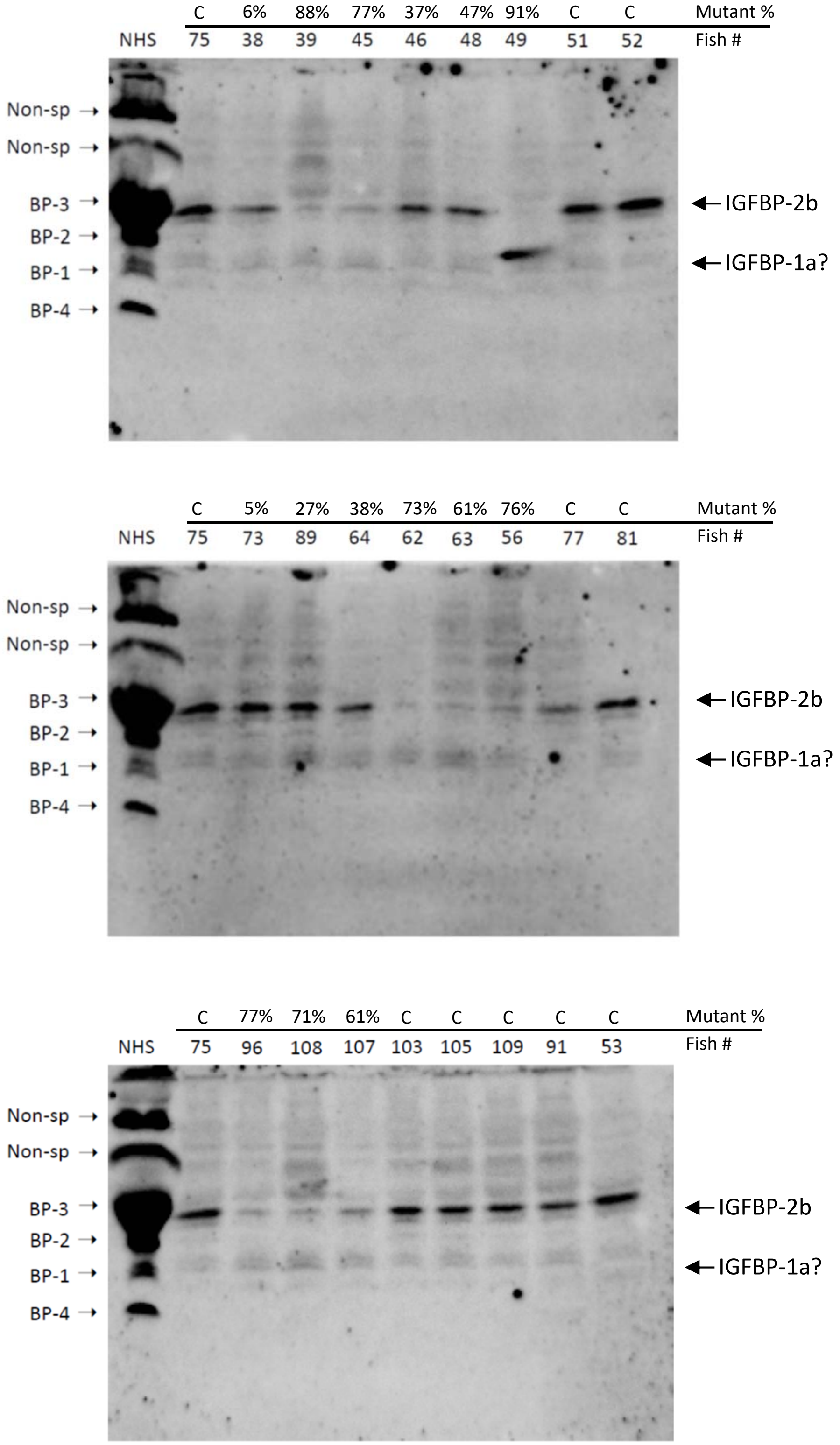

Supplement: Supplementary file 1 — Supplementary Files [file 41598_2018_34326_MOESM1_ESM.pdf]
